# A Structural Equation Modeling Approach to the Moderating Effect of Psychological Well-Being on Burnout and Depressive Symptoms Among Mental Health Professionals

**DOI:** 10.3390/healthcare14020284

**Published:** 2026-01-22

**Authors:** Fatih Bal, Hale A. Kahyaoğlu Çakmakci, İpek Okkay, Gülşen Filazoğlu Çokluk, Melek Süler

**Affiliations:** 1 Department of Psychology, Sakarya University, Sakarya 54187, Turkey; meleksuler@sakarya.edu.tr; 2 Department of Psychology, Atlas University, Istanbul 34403, Turkey; hale.cakmakci@atlas.edu.tr; 3 Department of Public Relations, Ayvansaray University, Istanbul 34087, Turkey; ipekokkay@yahoo.com; 4 Department of Psychology, Toros University, Mersin 33140, Turkey; gulsenfilazoglu@gmail.com

**Keywords:** burnout, depressive symptoms, psychological well-being, moderation, mental health professionals

## Abstract

**Background/Objectives:** This study investigates whether psychological well-being moderates the relationship between burnout and depressive symptoms among mental health professionals, who are frequently exposed to high emotional labor. **Methods:** A sample of 607 professionals—including psychologists, guidance counselors, social workers, and psychiatrists—working in public and private institutions in Turkey participated in the study. Data were collected online using the Maslach Burnout Inventory, the Psychological Well-Being Scale, and the Beck Depression Inventory–II. A latent interaction term between burnout and psychological well-being was tested using Structural Equation Modeling (SEM). **Results:** Burnout was positively associated with depressive symptoms (β = 0.37, *p* < 0.001), and psychological well-being showed a significant main effect on depressive symptoms (β = 0.26, *p* < 0.001). Importantly, the interaction between burnout and psychological well-being was significant (β = 0.20, *p* < 0.001), indicating that psychological well-being significantly moderates the relationship between burnout and depressive symptoms. Specifically, the positive interaction suggests that the association between burnout and depressive symptoms becomes stronger at higher levels of psychological well-being. The model explained 27% of the variance in depressive symptoms. **Conclusions:** These findings highlight the protective role of psychological well-being and suggest that interventions aimed at enhancing well-being may help reduce depressive symptoms among mental health professionals in high-stress environments.

## 1. Introduction

Mental health professionals are particularly vulnerable to intense emotional labor, high empathic demands, and chronic psychological stress inherent in their professional roles [1,2,3]. These occupation-specific stressors substantially increase the risk of adverse psychological outcomes, most notably burnout and depressive symptoms [4,5,6,7]. Evidence from a multinational longitudinal study conducted during the COVID-19 pandemic indicated a progressive deterioration in occupational well-being among mental health professionals, with younger and female practitioners being particularly affected [8]. Younger and female mental health professionals may be particularly vulnerable to occupational well-being deterioration due to increased work–family conflict, lower job control, limited organizational authority, and heightened emotional labor expectations. Early career professionals often face role ambiguity, performance pressure, and insufficient professional support, while female professionals are more likely to experience emotional exhaustion due to disproportionate caregiving responsibilities and societal role expectations. In addition, continuous exposure to clients’ trauma and emotional suffering places professionals at risk of compassion fatigue and secondary traumatic stress [9]. Insufficient institutional support and persistent work–life imbalance may further exacerbate burnout symptoms in healthcare settings [10].

Burnout is conceptualized as a psychological syndrome characterized by emotional, mental, and physical exhaustion resulting from prolonged exposure to occupational stressors, leading to diminished motivation, impaired well-being, and reduced professional efficacy [11]. One of the most frequently observed consequences of burnout is the development of depressive symptoms, including persistent low mood, fatigue, and loss of interest in both professional and personal domains [12]. Given the conceptual and empirical overlap between burnout and depression, identifying psychological resources that can mitigate or buffer this relationship is of particular importance. A systematic review and meta-analysis reported that approximately 40% of mental health professionals experience high levels of emotional exhaustion, underscoring the urgent need for effective preventive and supportive interventions in clinical work environments [13]. Beyond mental health outcomes, burnout has also been associated with long-term cardiovascular and physiological risks as well as increased hopelessness and suicidal ideation in high-stress professions such as medicine [14,15].

Psychological well-being (PWB) represents a key personal resource and is commonly defined as a multidimensional construct encompassing purpose in life, autonomy, environmental mastery, personal growth, self-acceptance, and positive interpersonal relationships [16,17]. Higher levels of psychological well-being have been consistently linked to greater resilience, more effective coping strategies, and reduced vulnerability to stress-related psychological distress [18,19]. Individuals with elevated PWB tend to maintain emotional stability and functional capacity even under demanding work conditions. Empirical evidence further indicates that while organizational risk factors, such as excessive administrative burden, poor communication, and workplace bullying, contribute to burnout, individual psychological resources—including psychological well-being—serve as important protective factors [20]. In healthcare contexts, psychological well-being has been shown to facilitate emotional regulation and protect against stress-related mental health problems [21].

From a theoretical perspective, Conservation of Resources (COR) theory provides a useful framework for understanding the role of psychological well-being in occupational stress processes [22]. COR theory posits that individuals strive to acquire, maintain, and protect valuable resources such as energy, emotional stability, and psychological strength. Burnout reflects a substantial depletion of these resources, whereas psychological well-being can function as a resource reservoir that mitigates the negative psychological consequences of resource loss [23]. COR theory has been widely applied in occupational health research to explain how emotional exhaustion and psychological distress emerge when personal and organizational resources are threatened or depleted [24].

Closely aligned with COR theory, the stress-buffering model offers a specific explanatory lens for examining moderation effects. According to this model, the impact of stressors (e.g., burnout) on psychological outcomes (e.g., depressive symptoms) depends on the availability of protective psychological resources, which can weaken or buffer adverse effects [25]. Despite its strong theoretical foundation, empirical research examining the moderating role of psychological well-being in the relationship between burnout and depressive symptoms remains limited, particularly among mental health professionals. Existing studies have predominantly examined burnout and psychological well-being as outcomes or as linearly related constructions, rather than testing psychological well-being as a moderator [26]. Moreover, empirical evidence from non-Western contexts, including Turkey, is notably scarce.

Against this backdrop, the present study aims to investigate the moderating role of psychological well-being in the relationship between burnout and depressive symptoms among mental health professionals. Specifically, it examines whether higher levels of psychological well-being attenuate the strength of the association between burnout and depressive symptoms. Grounded in the stress-buffering model and the Conservation of Resources theory, the study employs a structural equation modeling approach to test this moderation effect in a heterogeneous sample of psychologists, guidance counselors, social workers, and psychiatrists working in public and private institutions in Turkey. By addressing an underexplored research gap, this study aims to contribute to international literature while also providing context-specific insights that may inform mental health policy and intervention strategies aimed at reducing burnout and promoting psychological resilience among high-risk occupational groups.

## 2. Method

This study employed a quantitative, cross-sectional correlational design to examine the relationships among burnout, psychological well-being, and depressive symptoms in mental health professionals. Specifically, the study examined the direct relationship between burnout and depressive symptoms and investigated whether psychological well-being moderates this association.

Based on theoretical assumptions derived from the stress-buffering model and Conservation of Resources (COR) theory, it was hypothesized that higher levels of burnout would be associated with higher levels of depressive symptoms. Psychological well-being was conceptualized as a protective psychological resource expected to attenuate the strength of this association. Accordingly, a moderation model was specified in which psychological well-being interacts with burnout in predicting depressive symptoms.

To test the proposed hypotheses, a structural equation modeling (SEM) approach was adopted. The structural model included:

- The direct effect of burnout on depressive symptoms,
- the direct effect of psychological well-being on depressive symptoms, and
- an interaction term representing the moderating effect of psychological well-being on the relationship between burnout and depressive symptoms (Burnout × Psychological Well-Being).

All observed variables were standardized prior to analysis to facilitate the estimation of interaction effects and reduce multicollinearity. The interaction term was created following standard procedures for moderation analysis within SEM. The model was specified and estimated using IBM SPSS AMOS (Version 29; IBM Corp., Armonk, NY, USA).

**Figure 1:**
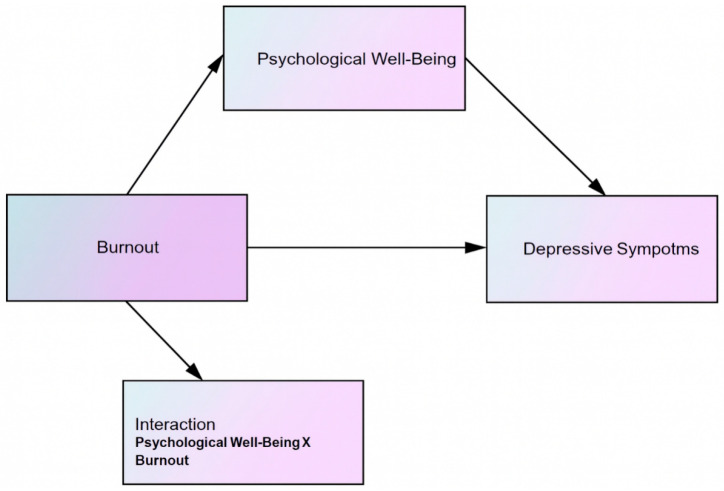
Symbolic Representation of the Research Model.

Model parameters were estimated using the maximum likelihood estimation (MLE) method. Model fit was evaluated using multiple goodness-of-fit indices: the chi-square to degrees of freedom ratio (χ^2^/df), the Comparative Fit Index (CFI), the Tucker–Lewis Index (TLI), the Root Mean Square Error of Approximation (RMSEA), and the Standardized Root Mean Square Residual (SRMR). These indices were interpreted according to commonly accepted cutoff criteria. A graphical representation of the proposed structural model is provided in Figure 1.

### 2.1. Participants

The study sample consisted of 607 mental health professionals (psychologists, guidance counselors, social workers, and psychiatrists) employed in public and private healthcare institutions across Turkey. Of the participants, 45.3% were female (n = 275) and 54.7% were male (n = 332). Regarding age, 46.6% were between 20 and 30 years old, 43.0% were between 31 and 45 years old, and 10.4% were 46 years or older. Regarding marital status, 83.7% of the participants were single (n = 508), and 16.3% were married (n = 99).

In terms of professional distribution, 50.9% were psychologists (n = 309), 33.8% were guidance counselors (n = 205), 9.9% were social workers (n = 60), and 5.4% were psychiatrists (n = 33). All participants had a minimum of one year of professional experience and were actively providing services to clients or patients at the time of data collection. Participation was voluntary.

### 2.2. Ethics

Prior to participation, all respondents provided informed consent. Ethical approval was obtained from the University Ethics Committee (Protocol No. 2025-01-78/1 January 2025). All procedures were conducted in accordance with the ethical principles outlined in the Declaration of Helsinki.

### 2.3. Data Collection Instruments

Maslach Burnout Inventory (MBI): Burnout was assessed using the Maslach Burnout Inventory, adapted into Turkish by Ergin [27,28]. The inventory comprises three subscales: emotional exhaustion, depersonalization, and reduced personal accomplishment. The Maslach Burnout Inventory consists of 22 items rated on a 5-point Likert scale (0 = never, 4 = always). A sample item is ‘I feel emotionally drained from my work.’ In the present study, a composite burnout score was used for the structural model. The scale demonstrated high internal consistency (Cronbach’s α = 0.89).

Psychological Well-Being Scale: Psychological well-being was measured using Ryff’s Psychological Well-Being Scale, adapted into Turkish by Telef [16,29]. The scale assesses six dimensions: autonomy, environmental mastery, personal growth, positive relations with others, purpose in life, and self-acceptance. The Psychological Well-Being Scale consists of 42 items rated on a 6-point Likert scale (1 = strongly disagree, 6 = strongly agree). A sample item is ‘I have a sense of direction and purpose in life.’ In the current study, the overall scale score was used, yielding strong internal consistency (Cronbach’s α = 0.90).

Beck Depression Inventory–II (BDI-II): Depressive symptoms were assessed using the Beck Depression Inventory–II, adapted into Turkish by Hisli [30,31]. The scale measures the severity of depressive symptoms, with higher scores indicating greater symptom severity. The Beck Depression Inventory–II consists of 21 items rated on a 4-point scale (0–3). A sample item is ‘I feel sad most of the time.’ The internal consistency coefficient in the present sample was 0.91. Based on established cut-off scores, the average depressive symptom level of the sample fell within the minimal to moderate range.

### 2.4. Procedure

Data were collected between January and March 2025 using an online survey platform. Participants received detailed information about the study objectives, confidentiality, and voluntary participation prior to providing electronic informed consent. Completion of the questionnaires required approximately 15–20 min. All procedures complied with the ethical standards of the 2013 revision of the Declaration of Helsinki.

### 2.5. Data Analysis

Data analyses were conducted using SPSS 29 and AMOS 29. Prior to hypothesis testing, the data were screened for normality, multicollinearity, and outliers. To test the moderating role of psychological well-being, an interaction term (Burnout × Psychological Well-Being) was computed and incorporated into the structural equation model. Structural Equation Modeling SEM (maximum likelihood estimation) was used to test direct and interaction effects. Statistical significance was set at *p* < 0.05. The interaction effect was further probed using simple slope analyses at low (−1 SD) and high (+1 SD) levels of psychological well-being.

## 3. Findings

Descriptive statistics for the main study variables are presented in Table 1. Overall, participants reported moderate levels of burnout and depressive symptoms, along with relatively high levels of psychological well-being. These descriptive results provide a contextual basis for interpreting the subsequent structural equation modeling results.

Table 1 presents the sociodemographic characteristics of the 607 mental health professionals included in the study. Of the participants, 45.3% were female (n = 275) and 54.7% were male (n = 332). Regarding age, 46.6% were between 20 and 30 years, 43.0% were aged 31–45, and 10.4% were 46 years or older, indicating that the sample primarily consisted of individuals in young and middle adulthood. With respect to marital status, 83.7% of the participants were single (n = 508), whereas 16.3% were married (n = 99). In terms of professional background, 50.9% of the sample were psychologists (n = 309), followed by guidance counselors (33.8%, n = 205), social workers (9.9%, n = 60), and psychiatrists (5.4%, n = 33). Overall, these results indicate that the study sample was heterogeneous and represented multiple professional groups within the field of mental health.

As illustrated in Table 2, the regression coefficients obtained from the structural equation model are presented, with the model examining the effects of burnout, psychological well-being, and their interaction on depressive symptoms. It was established that all structural paths were statistically significant (*p* < 0.001). The strongest predictor of depressive symptoms was found to be burnout (β = 0.367), indicating that higher levels of burnout are associated with greater depressive symptom severity.

Furthermore, the psychological well-being of the participants demonstrated a significant direct effect on their depressive symptoms (β = 0.263). Despite the positive standardized coefficient, which can be attributed to Z-score standardisation and the exclusion of reverse-coded depressive symptoms, the substantive interpretation indicates that higher psychological well-being corresponds to lower levels of depressive symptoms, thereby reflecting its overall protective main effect.

Of particular significance was the statistical significance of the interaction term between burnout and psychological well-being (β = 0.201, *p* < 0.001), which demonstrated a moderation effect. The positive interaction coefficient indicates that the association between burnout and depressive symptoms becomes stronger at higher levels of psychological well-being. This finding stands in opposition to a buffering interpretation, suggesting that psychological well-being amplifies rather than attenuates the impact of burnout on depressive symptoms. The model demonstrated moderate explanatory power, with an overall explanation of 27% of the variance in depressive symptoms (R^2^ = 0.27).

Table 3 presents the means, standard deviations, and Pearson correlation coefficients among burnout, psychological well-being, and the interaction term. Burnout (M = 15.03, SD = 3.35) and psychological well-being (M = 14.92, SD = 2.97) were moderately and negatively correlated (r = –0.33, *p* < 0.001), indicating that higher levels of psychological well-being were associated with lower levels of burnout.

**Figure 2:**
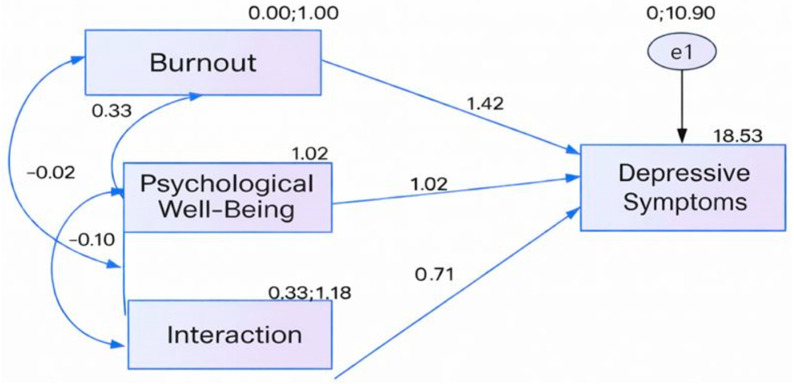
Structural Model Showing the Moderating Role of Psychological Well-being in the Relationship Between Burnout and Depressive Symptoms.

The correlations between the interaction term and burnout (r = −0.18, *p* < 0.001) as well as psychological well-being (r = −0.09, *p* < 0.05) were small in magnitude. These low correlations suggest that multicollinearity is minimal, supporting the statistical adequacy of the moderation analysis. The structural model illustrating the moderating role of psychological well-being is presented in Figure 2.

In the structural model developed using AMOS, the effects of burnout, psychological well-being, and their interaction on depressive symptoms were examined. All paths in the model were statistically significant at *p* < 0.001.

The direct effect of burnout on depressive symptoms was strong and positive (β = 0.367), indicating that higher burnout levels are associated with higher depressive symptom severity.

Psychological well-being also showed a significant direct effect (β = 0.263). Although the standardized coefficient appears positive, all variables were standardized using Z-scores, and depressive symptoms were not reverse-coded; therefore, higher psychological well-being corresponds to lower depressive symptoms in substantive terms, reflecting a protective effect.

The interaction term (Burnout × Psychological Well-Being) was significant (β = 0.201, *p* < 0.001), demonstrating that psychological well-being moderates the relationship between burnout and depressive symptoms. The interaction term (Burnout × Psychological Well-Being) was significant (β = 0.201, *p* < 0.001), demonstrating that psychological well-being moderates the relationship between burnout and depressive symptoms. Specifically, the positive interaction indicates that the association between burnout and depressive symptoms becomes stronger at higher levels of psychological well-being (i.e., an amplifying moderation pattern rather than a buffering one).

Correlation analyses supported the model assumptions. Burnout and psychological well-being were negatively correlated (r = −0.33, *p* < 0.001), and the correlations of the interaction term with burnout (r = −0.20) and with psychological well-being (r = −0.10) were weak but statistically significant, indicating minimal multicollinearity and supporting the validity of the moderation analysis.

**Figure 3:**
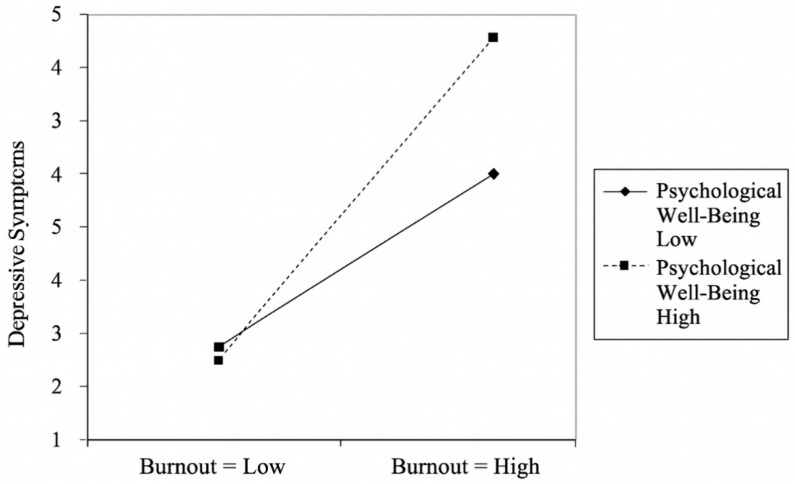
The Role of Psychological Well-Being as a Moderator Between Burnout and Depressive Symptoms.

The model explained 27% of the variance in depressive symptoms (R^2^ = 0.27), reflecting a moderate explanatory power [32]. These findings confirm the direct effect of burnout on depressive symptoms and highlight the protective, buffering role of psychological well-being. The moderating effect is illustrated in Figure 3.

In the interaction plot, the horizontal axis represents burnout levels (low to high), and the vertical axis represents depressive symptoms. The two lines depict low and high levels of psychological well-being. When burnout is low, depressive symptoms remain relatively low for both groups, with minimal differences. As burnout increases, depressive symptoms rise in both groups; however, the rate of increase differs depending on psychological well-being.

For individuals with high psychological well-being, depressive symptoms increase more steeply as burnout intensifies compared to those with low psychological well-being. This pattern indicates an amplifying (rather than buffering) moderating effect of psychological well-being on the burnout–depression relationship.

Although the standardized direct effect of psychological well-being on depressive symptoms appears positive (β = 0.263, *p* < 0.001), this should be interpreted in the context of Z-score standardization. Because depressive symptoms were not reverse-coded, higher psychological well-being corresponds to lower depressive symptoms, confirming its protective function in the model.

## 4. Discussion

This study examined the moderating role of psychological well-being in the relationship between burnout and depressive symptoms among mental health professionals. The results confirmed that burnout significantly predicts depressive symptoms. Importantly, psychological well-being significantly moderated this relationship (β = 0.201, *p* < 0.001); however, the positive interaction indicates an amplifying moderation pattern (i.e., the burnout–depression association becomes stronger at higher levels of psychological well-being), rather than a buffering effect.

Although the standardized regression coefficient of psychological well-being on depressive symptoms appears positive (β = 0.263), this reflects Z-score standardization. Since depressive symptoms were not reverse-coded, higher psychological well-being corresponds to lower depressive symptom levels, confirming its protective effect.

These findings align with Maslach and Jackson’s (1981) conceptualization of burnout, highlighting the link between emotional exhaustion, depersonalization, reduced personal accomplishment, and depressive symptoms [27]. The present model treated burnout as unidimensional; future research could explore its subdimensions separately to assess differential impacts or interactions with psychological well-being.

The results are consistent with Keyes and Ryff, suggesting that positive functioning and a sense of meaning contribute to resilience [17,33]. Elevated psychological well-being is associated with improved coping, emotional regulation, and reduced effects of occupational stressors [19,34]. Mindfulness- and resilience-based interventions in healthcare workers have been shown to reduce burnout and depressive symptoms [26,35,36]. These interventions may strengthen internal resources, buffer the effects of burnout, and enhance psychological safety and job satisfaction [37]. Contrary to the traditional stress-buffering hypothesis, the present findings indicate that psychological well-being amplifies the association between burnout and depressive symptoms. This suggests that individuals with higher psychological well-being may experience a sharper psychological contrast or resource depletion when exposed to intense burnout.

Graphical analyses of the interaction further illustrate that the increase in depressive symptoms due to burnout is less pronounced for those with higher psychological well-being. Conservation of Resources Theory supports this finding, emphasizing psychological well-being as a key protective resource [22].

The study has practical implications for both clinical practice and organizational policy. Interventions should not only reduce burnout but actively foster psychological well-being. Strategies may include mindfulness programs, supervision, psychoeducation, self-care modules, and resilience training, combined with organizational support and recognition to strengthen coping and reduce turnover. Regular well-being assessments integrated into occupational health monitoring may help detect early vulnerability; however, screening alone is insufficient without facilitated intervention [38].

Limitations include cross-sectional design, reliance on self-report measures, and potential bias due to online data collection. Z-score standardization improved comparability and reduced multicollinearity but may obscure clinical thresholds for depressive symptoms and burnout severity. The sample was heterogeneous yet unbalanced across professional groups, limiting subgroup analyses. Future research could use longitudinal or experimental designs, including multi-group SEM, explore profession- or gender-based moderation effects, and combine qualitative approaches to capture lived experiences. Further studies could also test additional well-being outcomes, such as life satisfaction, and investigate indirect or mediating pathways.

One possible explanation is that professionals with higher well-being and greater emotional investment in their work may experience a stronger sense of loss, disappointment, or incongruence when burnout occurs. This interpretation aligns with Conservation of Resources theory, which posits that individuals with greater resources may perceive resource loss more intensely. Promoting well-being while reducing burnout may be an effective strategy for supporting the psychological health and occupational functioning of mental health professionals.

## 5. Conclusions

In conclusion, the present study highlights the crucial role of psychological well-being in moderating the negative impact of burnout on depressive symptoms among mental health professionals. The findings indicate that psychological well-being significantly moderates the relationship between burnout and depressive symptoms; however, this moderation reflects an amplifying effect rather than a buffering one. This suggests that high psychological well-being does not necessarily protect individuals from the emotional consequences of burnout and may, under certain conditions, intensify depressive responses. These findings provide a strong empirical basis for implementing targeted interventions and policies at both individual and organizational levels. Integrating psychological well-being programs in workplace settings may not only reduce the risk of depression but also enhance resilience, job satisfaction, and long-term professional sustainability in emotionally demanding roles. Although burnout, psychological well-being, and depressive symptoms have been widely studied, empirical investigations testing psychological well-being as a moderator using latent interaction SEM models in non-Western samples remain scarce. This study extends existing literature by challenging the conventional buffering assumption and demonstrating an amplifying moderation pattern in a Turkish sample of mental health professionals.

## Figures and Tables

**Table 1:** Descriptive Analysis Results of Participants’ Sociodemographic Data.

Variables	Category	n	%
Gender	Female	275	45.3
	Male	332	54.7
Age	20–30	283	46.6
	31–45	261	43.0
	46 and above	63	10.4
Marital Status	Married	99	16.3
	Single	508	83.7
Profession	Psychologist	309	50.9
	Guidance Counselor	205	33.8
	Social Worker	60	9.9
	Psychiatrist	33	5.4
	Total	607	100.0

**Table 2:** Regression Coefficients for the Structural Equation Model.

Predictor Dependent Variable	Estimate	S.E.	C.R. p β	R^2^
Burnout → Depressive Symptoms	1.418	0.143	9.823 *** 0.367	0.270
Psychological Well-being → Depressive Symptoms	1.018	0.145	7.004 *** 0.263	
Interaction (Burnout × PWB) → Depressive Symptoms	0.714	0.126	5.682 *** 0.201	

Note: Estimate = unstandardized coefficient; S.E. = standard error; C.R. = critical ratio; β = standardized regression coefficient. *** *p* < 0.001.

**Table 3:** Means, Standard Deviations, and Correlations Among Burnout, Psychological Well-Being, and the Interaction Term.

Variable	M	SD	1	2	3
1. Burnout	15.03	3.35	−		
2. Psychological Well-Being	14.92	2.97	−0.33 ***	−	
3. Interaction (Burnout × PWB)	0.00	1.00	−0.18 ***	−0.09 *	−

* *p* < 0.05; *** *p* < 0.001.

## Data Availability

The data that support the findings of this study are available from the corresponding author upon reasonable request.
